# Using Sensory Evaluation to Determine the Highest Acceptable Concentration of Mango Seed Extract as Antibacterial and Antioxidant Agent in Fresh-Cut Mango

**DOI:** 10.3390/foods7080120

**Published:** 2018-07-30

**Authors:** Ariadna Thalia Bernal-Mercado, Jesus Fernando Ayala-Zavala, Manuel Reynaldo Cruz-Valenzuela, Gustavo A. Gonzalez-Aguilar, Filomena Nazzaro, Florinda Fratianni, Maria Raquel Alcantara de Miranda, Brenda A. Silva-Espinoza

**Affiliations:** 1 Centro de Investigacion en Alimentacion y Desarrollo, AC, Carretera a la Victoria km. 0.6, 83000 Hermosillo, Mexico; thalia.bernal@estudiantes.ciad.mx (A.T.B.-M.); jayala@ciad.mx (J.F.A.-Z.); reynaldo@ciad.mx (M.R.C.-V.); gustavo@ciad.mx (G.A.G.-A.); 2 ISA CNR, Institute Food Science, Via Roma 64, I-83100 Avellino, Italy; mena@isa.cnr.it (F.N.); fratianni@isa.cnr.it (F.F.); 3 Department of Biochemistry and Molecular Biology, Federal University of Ceará, Av. Mister Hull 2297, CEP 60440-554 Fortaleza, Brazil; rmiranda@ufc.br

**Keywords:** fresh-cut fruit, sensory attributes, antioxidant activity, phenolic compounds, antimicrobial activity, functional foods

## Abstract

Plant extracts have the potential to be used as food additives; however, their use have been limited by causing undesirable changes in the sensory attributes of foods. We characterized the mango seed extract as a preserving agent for fresh-cut mangoes. We established the maximum concentration of extract that, while increasing the antioxidant activity, and limiting microbial contamination of the fruit, did not negatively affect fruit sensory acceptability. The extract contained 277.4 g gallic acid equivalent (GAE)/kg dw (dry weight) of polyphenols and 143.7 g quercetin equivalent (QE)/kg dw of flavonoids. Antioxidant capacity values were 2034.1 and 4205.7 μmol Trolox equivalent (TE)/g against 2,2-diphenyl-1-picryl-hydrazyl (DPPH) and 2,2'-azino-bis(3-ethylbenzothiazoline-6-sulphonic acid) (ABTS) radicals, respectively. Chromatographic analysis revealed the presence of gallic and chlorogenic acids. The extract (16 g/L) inhibited the growth of *Escherichia coli*, *Salmonella* Typhimurium, *Staphylococcus aureus* and *Listeria monocytogenes*. The highest concentration with sensory acceptability was 6.25 g/L. At such concentration, the extract preserved fresh-cut fruits, increasing polyphenols (0.427 g GAE/kg fw (fresh weight)), flavonoid content (0.234 g QE/kg fw) and antioxidant activity (DPPH = 2.814 and ABTS = 0.551 mol TE/kg fw). It also reduced inoculated bacteria (range: 5.50 × 10^3^ to 1.44 × 10^5^ colony forming units (CFU)/g). These results showed the importance of considering consumer acceptability to determine the effective concentration of plant extracts as additives.

## 1. Introduction

The consumption of healthy and ready-to-eat foods has been increasing in recent years due to its great convenience for the population. In this sense, the food industry has promoted the consumption of fresh cut fruits. However, owing to their nature, they are highly perishable and it is necessary to use preservatives to lengthen their shelf life. Currently, consumers demand products with great nutritional value and without synthetic preservatives, so it is necessary to look for antimicrobial and antioxidants natural agents. Plant extracts are among the most studied ingredients to achieve this goal. Taking this into account, the use of agro-industrial byproducts has been proposed as an alternative due to them being a rich and economic source of polyphenol compounds with bioactive properties [1,2].

The seed, one of the byproducts of mango processing, shows strong antioxidant capacity and it contains a concentration of bioactive polyphenols higher than, not only mango pulp and peel, but also seeds from other plant species [3,4]. Major phenolic compounds found in mango seed extract are tannins, quercetin derivatives, gallic acid, ellagic acid and mangiferin [5]. These compounds are able to neutralize free radicals and inactivate microbial cells. Due to such properties, extracts from mango seeds and other byproducts could be added to several food matrices such as milk, cereal products and fresh-cut mango, with good results in terms of functionality and antimicrobial protection [6,7,8]; however, the sensory aspect has not especially been taken into account and mango seed constituents could have different effects on the sensory quality of foods. 

Phenolic compounds are the main constituents of the mango seed and such constituents can have predominantly sweet, bitter, pungent or astringent flavors [9]. Some plant phenolic compounds with high molecular weight, such as tannins, tend to be astringent, becoming unpleasant at high doses [9]. On the other hand, low molecular weight compounds, for instance gallic acid, are also considered as a sweetener [10,11]. This shows the possible impact of the use of mango seed extract in food matrices. In general, the application of plant extracts as food additives has been limited by causing undesirable changes in the sensory attributes of foods, which is an important attribute influencing the commercial success of the treated food [12,13,14]. Plant extracts may have tastes, colors and odors depending on their constituents and all such aspects should be taken into consideration before any use in the formulation of foods. However, few studies contemplate the acceptability of plant extracts to determine the right dose when used as food treatment. Therefore, the main goal of this study was to use sensory acceptability to determine the highest concentration of mango seed extract, rich in phenolic compounds, able to increase antioxidant capacity and reduce bacterial load of fresh-cut mango.

## 2. Materials and Methods 

### 2.1. Sample Characterization 

Mangoes “Haden” variety were obtained in a mature-green stage from a local market in Hermosillo, Sonora, Mexico. Fruits were selected according to homogenous color and free of physical damage. Mangoes were washed with chlorinated water (200 ppm) for 3 min and then, were allowed to dry at room temperature. The peel and seed were removed and the seed was stored at −20 °C for further use. The pulp was cut into small pieces and was physically and chemically characterized (Table 1) [15]. Color parameters (L, a*, b*) of mango slices were measured with a colorimeter (Minolta CR-300, Ramsey NJ, USA) and with those results hue angle and chroma were calculated. Total soluble solids were measured with a pocket refract PAL-1 (ATAGO, Tokyo, Japan), placing a drop of mango juice in the refractometer (previously calibrated with distilled water) and the °Brix were read. For the pH and titrable acidity, 10 g of mango pulp were diluted in 50 mL of distilled water. Then, the sample was filtered and titrated with NaOH 0.1 M in an automatic titrator (Mettler Toledo DL 28, Columbus, OH, USA). The results for titrable acidity were expressed as a percentage of citric acid. All determinations were made in triplicate. 

### 2.2. Preparation of Mango Seed Extracts

Mango seeds were chopped in small pieces (10 g) and put in a flask with 100 mL of ethanol: water (7:3). The sample was left to macerate in darkness for 10 days at 25 °C [8]. Subsequently, the extract was filtered through a Whatman No.1 (Springfield Mill, Maidstone Kent, UK). Then, the solvent was removed using a rotary evaporator (Büchi RE121, Brinkman, Flawil, Switzerland) and a water bath (Büchi 461, Brinkman, Flawil, Switzerland) with the followed conditions: reduced pressure, air as evaporation gas, bath temperature of 45 °C and a rotation of the evaporator of 60–80 rpm. Afterwards, the concentrated extract was subjected to an alkaline hydrolysis with NaOH 4 M for 4 h and then to an acid hydrolysis with HCl 4 M to adjust pH to 2.0. Finally, the extract was lyophilized and stored until use.

### 2.3. Identification and Quantification of Major Phenolic Compounds in Mango Seed Extract

Characterization of phenolic compounds was carried out using ultra-performance liquid chromatography (UPLC) with an ACQUITY Ultra Performance LC^TM^ system (Waters, Milford, MA, USA) linked to a PDA 2996 photodiode array detector (Waters). Ultraviolet-detection wavelength was set at 280 nm. Empower software (Waters) was used for controlling the instrument as well as for data acquisition and processing. The analysis was done at 30 °C using a reversed phase column (BEH C_18_ 1.7 µm, 2.1 × 100 mm; Waters). The mobile phase consisted of solvent A (7.5 mM acetic acid) and solvent B (acetonitrile) at a flow rate of 250 µL/min. Gradient elution was used starting at 5% solvent B for 0.8 min, 5–20% solvent B for 5.2 min, isocratic 20% solvent B for 0.5 min, 20–30% solvent B for 1 min, isocratic 30% solvent B for 0.2 min, 30–50% solvent B for 2.3 min, 50 –100% solvent B for 1 min, isocratic 100% solvent B for 1 min, and finally 100–5% solvent B for 0.5 min. At the end of this sequence, the column was equilibrated with initial conditions for 2.5 min. The pressure ranged from 6000 to 8000 psi during the chromatographic run and the effluent was introduced to a liquid chromatography detector (scanning range, 210–400 nm; resolution, 1.2 nm) and the injection volume was 10 µL [16]. Identification was made comparing UV spectra, using a database previously made with reference substances. Quantification was done using standard curves of the corresponding compound and reported as g of compound per kg dry weight (dw).

### 2.4. Total Phenolic Content of Mango Seed Extract

Total phenolic content was measured using the method of Singleton and Rossi [17] with some modifications. For the assay, 75 μL of Folin–Ciocalteu reagent (1:10) and 60 μL of 75 g/L Na_2_CO_3_ were mixed with 15 μL of sample. After incubation for 30 min in darkness, absorbance was measured at 765 nm using a FLUOstar Omega spectrophotometer (BMG Labtech Inc., Model Omega, Chicago, IL, USA). Total phenolic compounds were calculated using a standard curve of gallic acid and expressed as gram of gallic acid equivalents per kilogram of dry weight of extract (g GAE/kg dw). All samples were analyzed in triplicate. 

### 2.5. Total Flavonoid Content of Mango Seed Extract

Flavonoid content was determined using the method from Zhishen et al., [18] with some modifications. In darkness, 100 µL of sample were mixed with 430 µL of mixture A (1.8 mL of NaNO_2_ 50 g/L with 24 mL of distillate water) and incubated for 5 min. Then, 30 µL of AlCl_3_ (100 g/L) were added and incubated for one minute and after 440 µL of mixture B (12 mL of NaOH 1M with 14.4 mL of distillate water) were added. The absorbance of 150 µL of this solution was measured at 496 nm in a FLUOstar Omega spectrophotometer (BMG-Labtech Inc., Model Omega, Chicago, IL, USA). Total flavonoid compounds were calculated using a standard curve of quercetin and expressed as gram of quercetin equivalents per kilogram of dry weight of extract (g QE/kg dw). All samples were analyzed in triplicate. 

### 2.6. Antioxidant Activity of Mango Seed Extract by DPPH Radical Scavenging Assay

Total antioxidant activity was determined using the 2,2-diphenyl-1-picryl-hydrazyl (DPPH) method [19]. In darkness conditions, a stock solution was prepared mixing 2.5 mg of DPPH^•^ radical with 100 mL of pure methanol. Absorbance of DPPH^•^ solution was adjusted to 0.70 measured at 515 nm using a FLUOstar Omega spectrophotometer. Then, 140 μL of the radical solution and 10 μL of sample were mixed and incubated for 30 min and absorbance was read at 515 nm. Trolox was used as a standard and results were expressed as μmol trolox equivalents per gram of dried extract (μmol TE/g dw).

### 2.7. 2,2′-azino-bis(3-ethylbenzothiazoline-6-sulphonic acid) (ABTS) Radical Savenging Activity of Mango Seed Extract 

Trolox equivalent antioxidant capacity (TEAC) of samples was calculated using ABTS^•+^ [2,2′-azino-bis(3-ethylbenzothiazoline-6-sulfonic acid)] [20]. The ABTS^•+^ radical was generated in darkness mixing 5 mL of a solution of 7 mM ABTS^•+^ and 88 μL of 0.139 mM solution of K_2_S_2_O_8_. The radical solution was adjusted in methanol to have a final optical density (OD) of 0.7 measured at 754 nm. For the assay, 5 μL of sample and 245 μL of the ABTS solution were mixed and after 6 min OD was measured in a FLUOstar Omega spectrophotometer. Results were expressed as µmol of Trolox equivalents per gram of dried extract (µmol TE/g dw). All samples were analyzed in triplicate.

### 2.8. Antibacterial Activity

Antibacterial activity of mango seed extract was tested against *Escherichia coli* O157:H7 ATCC 43890, *Salmonella* enterica subsp. enterica serovar Typhimurium ATCC 14028, *Listeria monocytogenes* ATCC 7644, and *Staphylococcus aureus* ATCC 6538. The minimum inhibitory concentration (MIC) was determined using the micro-well dilution assay as previously described [21]. The range of mango seed extract concentrations evaluated was 0–20 g/L. Also, the minimal bactericidal concentration (MBC) was obtained to determine total inhibition of microbial growth by inoculating three concentrations above the MIC in Plate Count Agar and incubating at 37 °C for 24 h growth. Both MIC and MBC were expressed as g/L. In addition, growth curves of each bacterium at MIC values of mango seed extract were determined as previously indicated [21]. The experimental growth data for each bacterial strain were fitted to the Baranyi function using a complementary tool for Microsoft Excel (D-model, J. Baranyi, Institute of Food Research, Norwich, UK). The kinetic of growth parameters, including lag time (h), maximum specific rate (µmax, colony forming units (CFU)/h), and Y_max_ (CFU) for each growth curve, were calculated using the Baranyi function and the *R*^2^ was calculated. All analyses were done in triplicates. 

### 2.9. Sensory Evaluation of Fresh-Cut Mango Treated with Mango Seed Extract

To determine the maximum acceptable concentration of mango seed extract added to fresh-cut mangoes, a preliminary sensory analysis was done with 100 consumers (20–60 years old) who were not aware of the treatment. For this, mangoes cv “Haden” were sanitized and cut in cubes of 2 cm. Subsequently, cubes were immersed for 2 min within aqueous solution of seed extract at different levels (0.000, 1.580, 3.125, 6.250, 10.000 and 12.500 g/L); then, were allowed to dry and stored for 10 days at 5 °C. Sensory attributes were evaluated using a hedonic scale as ‘‘dislike very much”, “moderate dislike”, ‘‘neither like nor dislike”, “moderate like” and ‘‘like very much” (1 to 10, disliking to liking) [21]. Results were expressed as odor, color and taste acceptability. The maximum acceptable concentration determined was used to treat mango cubes. The sensory test was repeated to evaluate the effect of the treatment throughout the storage time (0, 5 and 10 days at 5 °C) with 100 consumers (20–60 years old), who were not aware of the treatment. 

### 2.10. Phenolic Content and Antioxidant Activity of Fresh-Cut Mango Treated with Mango Seed Extract

We assessed total phenol and flavonoid content and antioxidant activity of stored (10 days at 5 °C) fresh-cut mangoes treated or not with 6.25 g/L of mango seed extract. Sample tissue (10 g) was homogenized in 15 mL of methanol (80%), sonicated 30 min at 1 °C (Bransonic Ultrasonic Co., Model 2210, Danbury, CT, USA), centrifuged at 1200 *g* for 15 min at 4 °C (Allegra 64R, Beckman Coulter Centrifuge, Palo Alto, CA, USA) and filtered. This was repeated twice with 10 mL of methanol (80%) and the volume of the three supernatants were pooled and brought up to 40 mL [8]. Total phenolic, flavonoid content and antioxidant capacity (DPPH and ABTS) were measured from this solution as described in preceding sections. All analyses were done in triplicate.

### 2.11. Bacterial Load Reduction in Fresh-Cut Mango Treated with Mango Seed Extract

Mangoes were sanitized and chopped in cubes of 2 cm. Samples (10 g) were inoculated by immersion during 2 min in different solutions of bacteria (*E. coli*, *S.* Typhimurium, *L. monocytogenes* and *S. aureus* with an inoculum of 1 × 10^6^ CFU/mL, respectively) and were dried for 30 min in a biosafety cabinet (Esco II Airstream, Horsham, PA, USA) [21]. The initial load of each inoculated bacteria was determined. Then, the samples were immersed into a solution of mango seed extract at 6.25 g/L. Also, mangoes were dipped in only water without the extract and such samples were used as the control. Samples were left to dry during 30 min. Bacterial loads were determined on Plate Count Agar and results were expressed as colony forming units per gram of fruit (CFU/g). 

### 2.12. Statistical Analysis

A complete randomized design was done for all experiments. The effect of mango seed extract was tested on the kinetic parameters of bacteria growth (lag phase, µ_max_ and Y_max_). Also, the effect of mango seed extract on sensory acceptability, antioxidant activity and bacterial load reduction of fresh-cut mango was tested. Analysis of variance (ANOVA) was done (*p* < 0.05) to estimate significant differences between treatments and Tukey’s mean test was used for comparison (*p* < 0.05) using the software NCSS 2007 (NCSS Statistical Software, Kaysville, UT, USA). 

## 3. Results and Discussions

### 3.1. Phenolic Compounds, Antioxidant and Antimicrobial Activity of Mango Seed Extract

**Figure 1:**
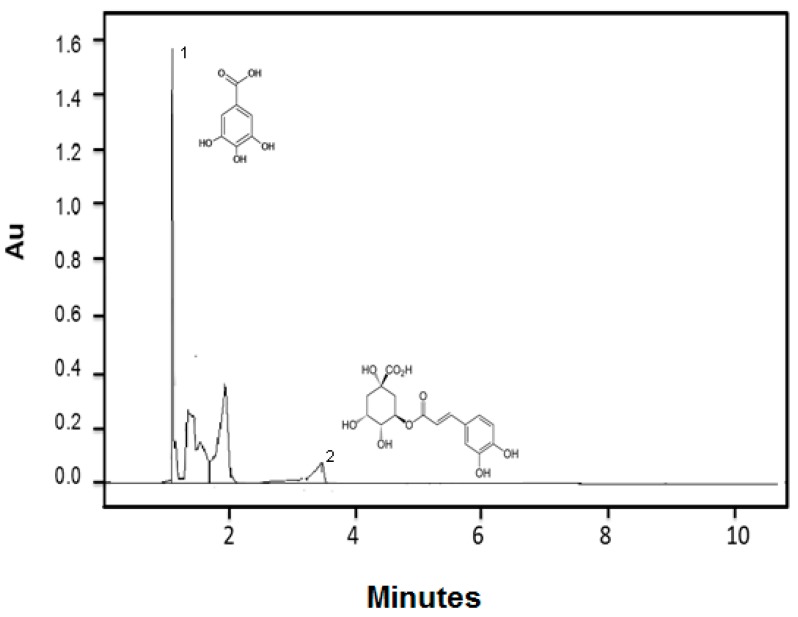
Ultra-high-performance liquid chromatography–diode array detector (UPLC-DAD) chromatogram showing phenolic compounds identified in mango seed extract. ^1.^Gallic acid. ^2.^Chlorogenic acid.

Mango seed extract showed a total phenolic and flavonoid content of 277.4 ± 0.75 g GAE/kg dw and 143.7 ± 1.86 g QE/kg dw, respectively. The main phenolic constituents identified in the seed extract were gallic (0.160 g/kg dw) and chlorogenic (0.100 g/kg dw) acids (Figure 1). Accordingly, antioxidant capacity values were 2034.1 ± 1.07 μmol TE/g and 4205.7 ± 13.15 μmol TE/g against DPPH and ABTS radicals, respectively. The studied extract showed two dietary phenolic compounds with antioxidant and antibacterial properties [4]. Compared to previous studies, Dorta et al., [5] showed that antioxidant activity of ethanolic extract from mango var. Keitt seeds against DPPH and ABTS radicals were lower than those reported here. Furthermore, the ethanolic extract from mango var. Haden seeds showed a similar flavonoid content (164.6 g QE/kg) and a higher phenolic content (875.1 g GAE/kg) than the extract reported in this study [4]. In general, mangiferin, isomangiferin, homomangiferin, quercetin, kaempferol, methyl gallate, ethyl gallate, galloyl glucose, penta-O-galloyl-glucoside, methyl gallate ester, anthocyanins and gallic, protocatechuic, ferulic, caffeic, coumaric, ellagic, 4-caffeoylquinic acids are reported in mango seeds from several varieties [5,22]. Abdalla et al., [23] reported that methanolic extracts obtained from a mix of Egyptian mango seeds contained tannins (0.207 g/kg dw), vanillin (0.202 g/kg dw) and mangiferin (0.420 g/kg dw), as well as gallic (0.600 g/kg dw), *p*-coumaric (0.126 g/kg dw), caffeic (0.770 g/kg dw), ferulic (0.104 g/kg dw) and cinnamic acids (0.112 g/kg dw). 

In addition, mango seed extract was able to inhibit *E. coli*, *S.* Typhimurium, *L. monocytogenes* and *S. aureus* at a MIC of 16 g/L and a MBC of 18 g/L for Gram-negative and 16 g/L for Gram-positive bacteria. Exposure to mango seed extract affected all bacterial growth parameters (Table 2). An extract concentration of 16 g/L significantly extended the lag phase (8.4 h) of *E. coli* and decreased both µ_max_ (68.3%) and Y_max_ (39%) when compared to control. Kinetic parameters of *S*. Typhimurium were also affected at 16 g/L, with an extension of lag phase (10.5 h) and reduction of µ_max_ (65.7%) and Y_max_ (39%). Similarly, the seed extract at 16 g/L extended the lag phase (12.5 h) and decreased µ_max_ (56.3%) and Y_max_ (56.6%) of *S. aureus*. Regard of *L. monocytogenes*, the extract at 16 g/L was able to extent the lag phase to 24 h and no changes were observed in µ_max_ nor Y_max_. 

It has been reported that Gram-negative bacteria are more resistant to mango seed extract [22] compared to the Gram-positives ones, because they have an outer lipid membrane covering the entire cell that hinders the effect of antibacterial agents. In addition, membrane disruption is one of the antibacterial mechanisms of phenolic compounds [24]; thus, a double membrane could be harder to attack. Our results show the efficacy of mango seed extract to affect different phases of bacterial growth. An increase in the lag phase indicates that the extract affected bacteria adaptation; this could be attributed to an inhibition that could affect nutrient uptake or the activity of viable enzymes for microbial cell physiology. It has been suggested that phenolic acids and flavonoids can chelate metals that are necessary for enzyme activity in metabolic processes giving rise to microbial growth [25]. Also, phenolic compounds such as gallic acid may cause irreversible modifications to bacterial membrane properties (charges and intra and extracellular permeability) through hydrophobicity changes, decreases in negative charges of surface and rupture or pore formation, with consequent leakage of essential intracellular components [26].

Previous studies showed that mango extracts contained compounds with higher molecular weight such as tannins, not detected here and that only simple compounds such as gallic acid were identified (Figure 1). This might be attributed to the hydrolysis of phenols, with formation of simpler compounds [27]. Soong and Barlow [28] found that after thermal and acid hydrolysis treatment, methanolic extract from seeds showed higher concentration of gallic and ellagic acids than seeds without hydrolysis, contributing to a higher antioxidant activity. This suggests that conjugated compounds may be liberated using acid hydrolysis and their free forms may be more potent as antioxidants as observed in this study. 

### 3.2. Sensory Evaluation of Fresh-Cut Mango Treated with Mango Seed Extract

**Figure 2:**
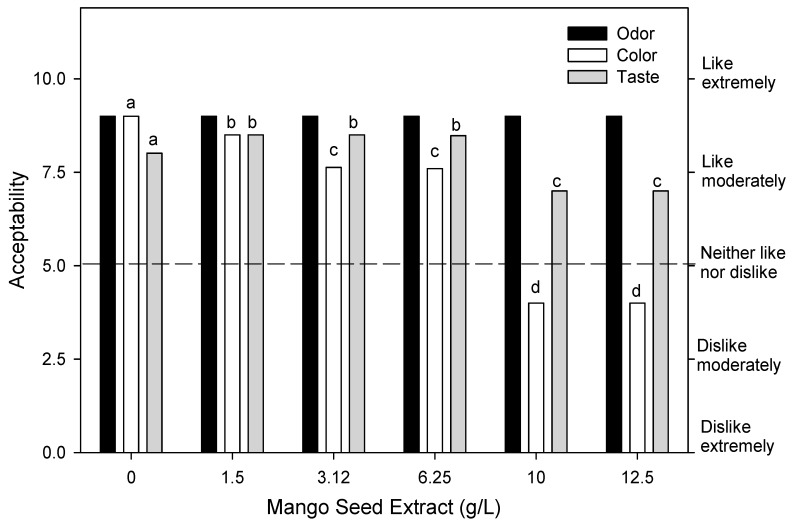
Liking level of odor, color, and taste of fresh-cut mango fruits treated with different mango seed extract concentrations. Different letters in the same parameter evaluated means significant difference (*p* < 0.05).

The effect of mango seed extract on overall acceptance of fresh-cut mango storage for 10 days at 5 °C is showed in Figure 2. Odor acceptance did not show differences (*p* > 0.05) between treatments and consumers scored “liked very much” the odor of treated and control fruits. Color acceptance by consumers differed significantly among treatments. The panelists scored better on the color of the control fruits. Color acceptance of fruits treated with 1.5, 3.12 and 6.25 g/L showed moderate liking levels; while the fruits treated with the highest concentrations (10 and 12 g/L) were not pleasant for the panelists. Taste acceptability was positively affected using 1.5, 3.12 and 6.25 g/L of seed extract, and showed higher liking levels respect to the control (*p* < 0.05). However, all treatments were scored as “moderate like”. According to these results, 6.25 g/L of mango seed extract was selected as the maximum acceptable concentration that did not affect odor, color and taste of fresh-cut mangoes. 

**Figure 3:**
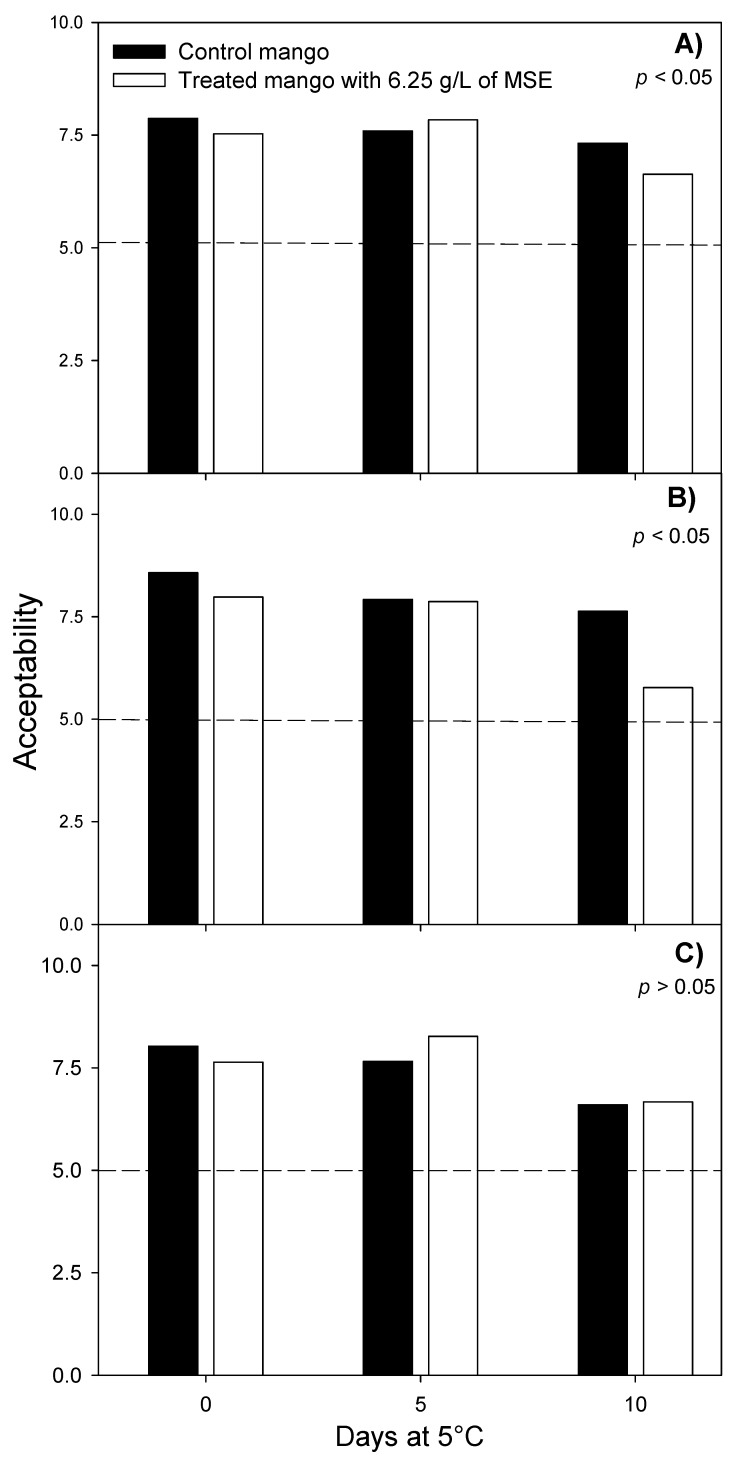
Effect of 6.25 g/L of mango seed extract (MSE) on the acceptability of (**A**) odor, (**B**) color and (**C**) taste of fresh-cut mango stored at 5 °C for 10 days (Scale 10 = like extremely, 7.5 = like moderately, 5 = neither like nor dislike, 2.5 = dislike moderately, 0 = dislike extremely). *p* < 0.05 means significant difference between control and treatment.

This concentration was selected for further sensory evaluation (*n* = 100), and to evaluate the antioxidant and antibacterial activity of fresh-cut mangoes treated during storage. The effect of mango seed extract (6.25 g/L) on the total acceptability of the fresh-cut mangoes stored for 10 days at 5 °C is showed in Figure 3. Sensory analysis showed a significant difference (*p* < 0.05) between the color of control and treated fresh-cut mangoes at 10 d of storage at 5 °C. Despite this significant difference, the level of color acceptance of treated fruits was scored as “moderate like” by consumers. However, no differences were found (*p* > 0.05) in odor and taste attributes between treated and control fruits, being both scored as “moderate like”. Mango seed extract at 6.25 g/L did not impact negatively on the taste of fresh-cut mango, and consumers expressed a better taste in treated fresh-cut mangoes indeed. 

Mango seed extract exhibited potential as a food additive at 6.25 g/L with overall good consumer acceptance. Mango seed extract did not affect fresh-cut fruit odor due to its non-volatile composition, which is usually responsible for aroma and odor of many foodstuffs. On the other hand, the taste of treated fresh-cut mango was not affected, probably because the main component of mango seed extract, which is gallic acid, is described as a sweetener [10]. Such a compound gives a long-term sweetness, and it does not cause aftertaste; its effect appears to be more powerful and lasting. This might explain why panelists experienced a sweet and better taste in treated mango. In addition, the use of 6.25 g/L did not reduce acceptability of fruit color, although the fruit was slightly darker. A similar effect was previously reported, the extract of green tea at concentrations higher than 5 g/L added to increase the antioxidant content of fresh-cut lettuce, caused a darkening in the sample [29]. The authors proposed that the increment in browning of lettuces probably was due to the high polyphenol content and to the activity of polyphenol oxidase enzyme in samples [29]. 

Previous reports described the addition of fruit by-product extracts to different food matrices. Ajila et al. [30] incorporated mango peel powder at different concentrations (25, 50 and 75 g/kg) to formulate functional macaroni. Using sensory analysis, panelists accepted macaroni containing up to 50 g/kg with reference to color, taste, and texture. Similarly, the addition of the banana peel extract at 5 g/L to orange gave rise to a product accepted by consumers by taste, odor, and color, but undesirable changes in mouth sensations and color were detected at concentrations of 10 g/L [31]. Our results may be explained by the nature of by-product extracts; for instance, mango peel extract is rich in tannins, known to have an astringent flavor, which can result in being unpleasant to the consumers at high concentrations. On the other hand, phenolic compounds at low concentrations do not affect flavor. Therefore, identifying the most appropriate concentration of the extract plays an important role assuring consumer acceptability.

### 3.3. Phenolic Compounds and Antioxidant Capacity of Fresh-Cut Mango Treated with Mango Seed Extract

**Figure 4:**
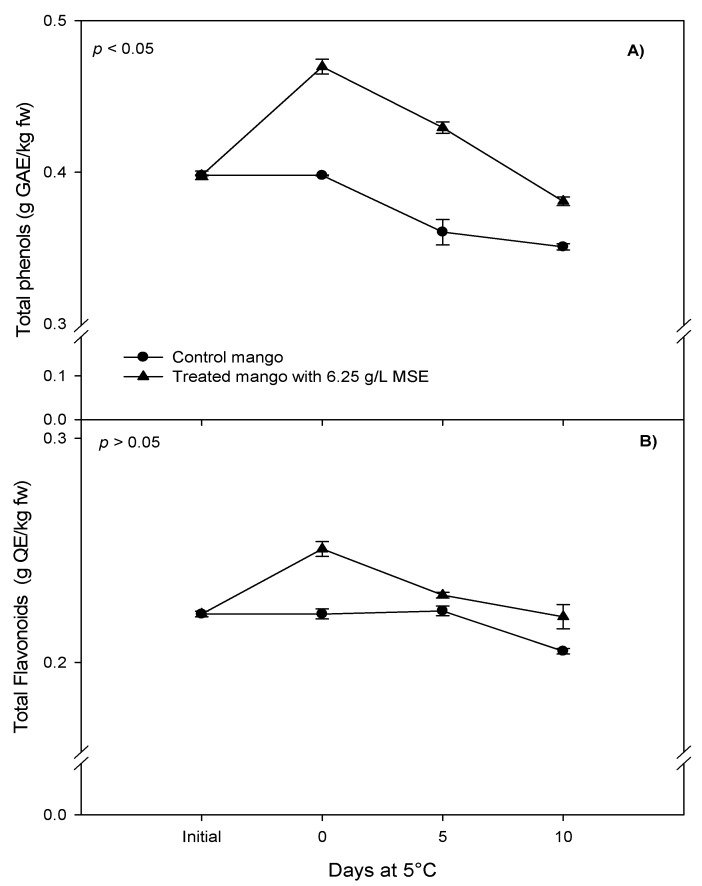
Effect of 6.25 g/L of mango seed extract (MSE) in the total (**A**) phenol and (**B**) flavonoid content of fresh-cut mango stored at 5 °C for 10 days. *p* < 0.05 means significant difference between control and treatment. Means ± standard error.

**Figure 5:**
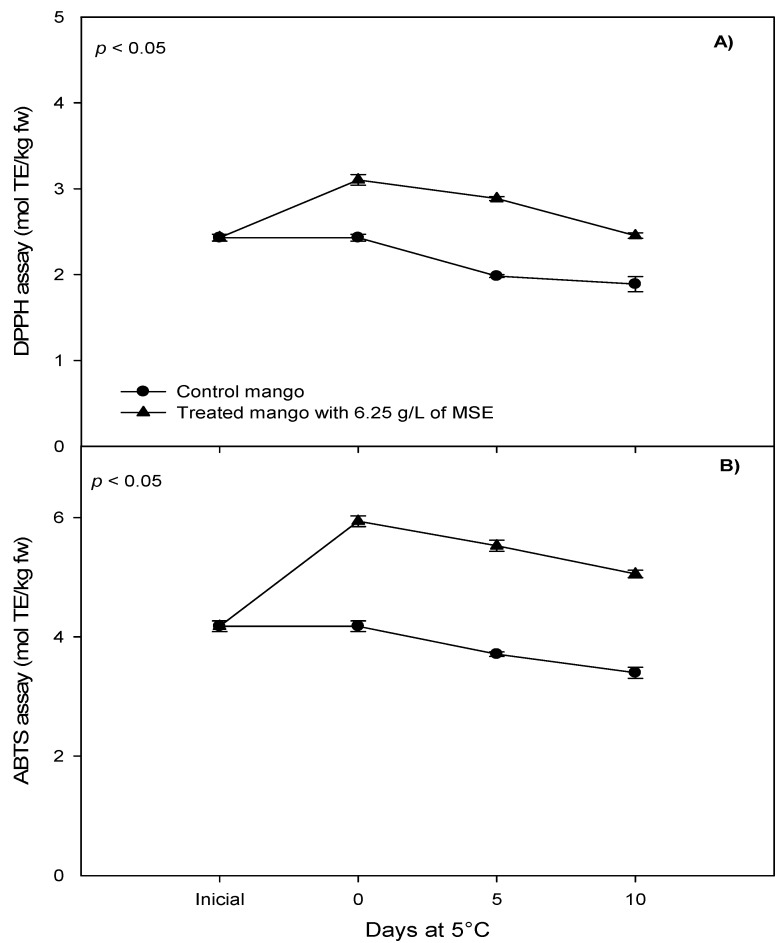
Effect of 6.25 g/L of mango seed extract (MSE) in the antioxidant capacity for the (**A**) 2,2-diphenyl-1-picryl-hydrazyl DPPH assay and (**B**) 2,2′-azino-bis(3-ethylbenzothiazoline-6-sulphonic acid) (ABTS) assay of fresh-cut mango stored at 5 °C for 10 days. *p* < 0.05 means significant difference between control and treatment. Means ± standard error.

A significant effect of the treatment (6.25 g/L of seed extract) was observed on total phenolics, flavonoid content and on the antioxidant capacity of fresh-cut mangoes (Figure 4 and Figure 5). Overall, the extract of mango seed (6.25 g/L) increased the total polyphenols content of fresh-cut mango as respects to the control (0.427 g GAE/kg fw and 0.358 g GAE/kg fw, respectively) (*p* < 0.05). A similar result was observed with respect to the flavonoid content, which resulted higher in treated mango at day 0 than control fruits. At the end of storage, treated fruit retained its flavonoids level, in contrast to the control; this indicated that mango seed extract might have contributed to the preservation of flavonoid levels. Treated mangoes showed higher antioxidant activity than the control at day 0 and at the end of the storage, and an increment in antioxidant capacity during storage. Regarding the global effect, mango seed extract at 6.15 g/L increased the capacity to inhibit DPPH radical (2.814 mol Trolox equivalents (TE)/kg fw), as compared to the control (2.10 mol TE/kg fw) (*p* < 0.05). Similar results were observed, using ABTS as compared to the control (0.551 and 3.763 mol TE/kg fw, respectively) (*p* < 0.05). Treatment with mango seed extract, increasing the total polyphenols and strengthening the antioxidant capacity of fresh-cut mango, places this as a potential candidate for the role of functional food with beneficial effects on human health.

The increase of antioxidant capacity exhibited by treated fruit could be justified simply by the addition of antioxidants taking place from the mango seed extract. A similar approach was followed in other studies: mango seed extract added to fresh-cut mango var. Haden increased polyphenols (7.4 times) and flavonoids (3.1 times) and the antioxidant capacity using DPPH, TEAC and oxygen radical absorbance capacity (ORAC) (2.9, 2.3 and 2.8 times, respectively) [8]; however, no sensory test was carried out to evaluate the acceptability by the consumers. On the other hand, macaroni treated with mango peel extract (5 g/kg) were defined as sensory acceptable and showed higher amounts of polyphenols (0.46 to 1.80 g/kg) and carotenoids (0.5 to 0.84 g/kg) [30]. This highlights the importance of evaluating the appropriate extract concentration of a natural preserving agent that could be capable of guaranteeing sensory acceptance and concurrently, to assure the presence of higher phenolic and antioxidant content in the treated product. 

### 3.4. Bacterial Load of Fresh-Cut Mango Treated with Mango Seed Extract

Mango seed extract at 6.25 g/L reduced (*p* < 0.05) inoculated bacteria in fresh-cut fruit. In fact, treated fruit showed 8.50 × 10^3^ CFU/g of *E. coli*; on the contrary, control fruits exhibited 1.40 × 10^4^ CFU/g. A similar effect was observed also when treated fruit was inoculated with *S.* Typhimurium (2.40 × 10^4^ CFU/g) compared to the control, which contained higher bacterial load (6.30 × 10^4^ CFU/g). Mango seed extract was also effective in reducing *S. aureus* compared to the control (4.50 × 10^4^ CFU/g and 9.50 × 10^4^ CFU/g, respectively). Mango inoculated with *L. monocytogenes* showed a value of 1.68 × 10^5^ CFU/g, treatment with mango seed extract determined a decrease of 2.40 × 10^4^ CFU/g. Through the in vitro assays, mango seed extract proved to be more effective against Gram-positive than Gram-negative bacteria. Such results show the benefits of using mango seed extract to reduce the pathogenic bacterial load of fresh-cut mango without affecting its sensory acceptability. 

In other studies, mango seed extracts reduced 80% of aerobic mesophilic and 97% of total fungi in fresh-cut mangoes stored for 15 days at 5 °C [8]; however, even in this case the microbiological studies were associated with assessments on sensory acceptability by a panel of consumers. On the other hand, Abdalla et al., [7] reported that the addition of 3 g/L of mango seed extract to pasteurized cow milk reduced aerobic mesophilic (6.3 log CFU/g) and total coliform (6.32 log CFU/g) counts and was sensory accepted by consumers. This indicated that it is possible to use plant extracts at certain concentrations that are sensory acceptable and provides a positive effect on the increment of antioxidant capacity and reducing bacterial load.

## 4. Conclusions

Sensory attributes of fresh-cut mangoes treated with mango seed extract at 6.25 g/L were rated acceptable by consumers. Moreover, at this concentration, mangoes showed a higher phenolic compound and antioxidant capacity and a lower bacterial load than untreated fruit. This study showed the benefits of mango seed extract as an antibacterial, antioxidant and sensory additive to use it in the formulation of functional foods.

## Figures and Tables

**Table 1:** Physicochemical characterization of mangoes used in the experiments (*n* = 3).

Parameters	Values
Total soluble solids (°Brix)	10.43 ± 0.13 *
pH	3.82 ± 0.07
Titrable acidity (%)	0.80 ± 0.004
Pulp color	
L	72.15 ± 0.75
Hue angle	85.82 ± 0.21
*Chroma*	62.24 ± 0.42

* Mean ± standard error.

**Table 2:** Effect of different concentrations of mango seed extract (MSE) on growth parameters of pathogenic bacteria.

Bacteria	MSE Treatment (g/L)	Lag (h)	μ_max_ (CFU/h)	Y_max_ (log CFU)	*R* ^2^
*E.coli*	0	2.5 ^a,^*	0.41 ^a^	10.50 ^a^	0.97
	8	3.0 ^a^	0.22 ^ab^	9.70 ^b^	0.94
	16	8.4 ^b^	0.13 ^b^	6.40 ^c^	0.98
*S.* Typhimurium	0	3.0 ^a^	0.35 ^a^	9.97 ^a^	0.98
	8	5.0 ^b^	0.28 ^a^	9.32 ^b^	0.98
	16	10.5 ^c^	0.12 ^b^	6.09 ^c^	0.97
*L. monocytogenes*	0	0.0 ^a^	0.26 ^a^	9.50 ^a^	0.98
	8	11.0 ^b^	0.34 ^a^	8.90 ^b^	0.98
	16	24.0 ^c^	0.00 ^b^	0.00 ^c^	ND
*S. aureus*	0	2.5 ^a^	0.48 ^a^	14.07 ^a^	0.98
	8	0.0 ^b^	0.40 ^a^	12.86 ^b^	0.99
	16	12.5 ^c^	0.21 ^b^	6.11 ^c^	0.98

* Different letters indicate that growth parameters are different between MSE concentrations for each bacteria (*p* < 0.05), for example, a value with an ^a^ is different to ^b^ and ^c^, and a value with a ^b^ is different to ^c^.
